# Mathematical Methods for Images and Surfaces

**DOI:** 10.1155/2010/918467

**Published:** 2011-03-31

**Authors:** Guowei Wei, Lalita Udpa, Yang Wang, Shan Zhao

**Affiliations:** ^1^Department of Mathematics, Michigan State University, East Lansing, MI 48824, USA; ^2^Department of Electrical and Computer Engineering, Michigan State University, East Lansing, MI 48824, USA; ^3^Department of Mathematics, University of Alabama, Tuscaloosa, AL 35487, USA

The last two decades have witnessed a greatly increasing interest in mathematical methods for images and surfaces that are originated from biomedical and biological applications. The research problems have necessitated the development of mathematical theories of wavelets, frames, harmonic analysis, geometric flows, differential geometry, topology statistical methods, machine learning, and so forth. This theoretical development is enhanced by the related numerical algorithm development and modeling development. Currently, mean curvature flow, Willmore flow, level set, generalized Laplace-Beltrami operator are commonly used mathematical techniques for the analysis of biomedical images and for the generation of biomolecular surfaces. Additionally, wavelets, frames, compressive sensing, and harmonic analysis are popular tools for biomedical visualization and image processing. Moreover, differential geometry, topology, and geometric measure theory are powerful approaches for the multiscale modeling of biomolecular structure, dynamics, and transport. Finally, persistently stable manifold, topological invariant, Euler characteristic, Frenet frame, and machine learning are vital to the dimensionality reduction of extremely massive biomedical and biomolecular data. These ideas have been successfully paired with current investigation and discovery in biomedical and biological systems. However, many mathematical challenges remain in image and surface analysis, such as the well-posedness of mathematical models under physical and biological constraints, maximum-minimum principle for high-order geometric evolution equations, numerical analysis of multiply coupled partial differential equations, effectiveness of approximation theory, and the modeling of complex biomolecular phenomena. This special issue was called to address mathematical difficulties and challenges in image and surface analysis. It consists of seventeen research papers. 

The first paper presents octahedral symmetrical adapted functions (OSAFs) for three-dimensional (3D) reconstruction of macromolecule assembles. The authors employed two biomolecules, namely, heat shock protein Degp24 and the Red-cell L Ferritin, to verify the feasibility and advantages of their OSAF method. Their results indicate that the OSAF method is feasible and efficient for reconstructing structures of macromolecules.

The second paper outlines statistical evaluations of the reproducibility and reliability of 3-Tesla high-resolution magnetization transfer brain images, which are very promising for early detection and monitoring of subtle brain changes. This paper analyzes 9 healthy subjects acquired from 12 brain regions of interest. It computes Spearman's correlation coefficient, coefficient of variation, and intraclass correlation coefficient. Multivariate mixed-effects regression models are built, and sensitivity analysis of various model specifications is carried out. 

The third paper investigates myocardial deformation and strain using suitably encoded cine MRI that admits disambiguation of material motion. The authors proposed a new method to obtain a robust estimation of the velocity gradient tensor field, which determines the time evolution of the deformation tensor. A first-order ordinary differential equation is solved for induced Lagrangian strain tensor field, yielding results that are typical for healthy volunteers.

The fourth paper introduces a differential geometry-based multiscale paradigm to model complex biomolecule systems. Geometric flows and related techniques have been widely used in image analysis and surface modeling. However, they have not been used for multiscale analysis. The authors proposed a novel multiscale approach by using geometric flows and variation formalism. Applications are considered for virus surface formation and evolution. 

The fifth paper discusses size functions for the morphological analysis and classification of melanocytic lesions. The proposed approach is based on the qualitative assessment of asymmetry via halving images. With appropriate thresholds, 977 clinic images are analyzed in terms of color, mass distribution, and boundary. Cross-validation of these images is also performed. 

The sixth paper presents a fast and parameterizable algorithm to represent molecular surfaces with good visual quality. This approach is based on isosurfacing a filtered electron density map, which is the result of the maximum of Gaussian functions placed around atom centers. The map visual quality is improved by filtering the Fourier Transform of the density map. The marching cubes algorithm is used to generate the molecular surface mesh after the inverse Fourier transform.

The seventh paper offers the principal component analysis of the lateral distribution of fluorescent lipid in binary-constituent lipid monolayers. The mixture of fluorescent and nonfluorescent lipids is approximated as the linear combination of only two principal vectors. The proposed method allows massive data compression, enables significantly enhanced resolution of lipid lateral organizational changes, and provides characterization of lipid lateral interactions. 

The eighth paper provides independent factor analysis to blindly separate hemodynamics from magnetic resonance perfusion brain images. Temporal signal changes on brain artery, gray matter, white matter, vein and sinus, and choroid plexus manifest distinct blood-supply patterns for the profound analysis of cerebral hemodynamics. The signal-time curves and the averaged signal time curve on the segmented arterial area were used to calculate the relative cerebral blood volume, relative cerebral blood flow, and mean transit time. Results are compared with those in the literature.

The ninth paper investigates photon transport in the platform of molecular optical simulation environment which represents biological tissues and free space for optical imaging. Monte Carlo method and the hybrid radiosity-radiance theorem are used for the simulation in parallel setting. The simulation results are compared with Tracepro, simplified spherical harmonics method, and physical measurement to validate the performance of the proposed method in terms of accuracy and efficiency.

The tenth paper applies a multilevel space-time aggregation method for segmenting live cell bright field microscope images. The present approach is modified from “segmentation by weighted aggregation” technique, an algebraic multigrid method. A new scale-invariant “saliency measure” is proposed. The authors provided preliminary results for applying the multilevel aggregation algorithm in space time to temporal sequences of microscope images. Discussions are given to the advantages and drawbacks of the space-time aggregation approach for cell segmentation and tracking as well as future application of the method. 

The eleventh paper highlights existing and newly developed feature detection algorithms for preprocessing proteomic data. These detection algorithms are particularly interesting because of the increased computational speed required by subsequent calculations of protein changes potentially associated with disease. Results are obtained via all gel-based and nongel-based methods. The present approaches provide preprocessed data for further statistical analysis.

The twelfth paper offers the method of weighted minimum-norm least squares for the determination of lateral modulation apodization functions. The author designs Gaussian-type point spread functions with lateral modulation to validate the proposed method. Results obtained with the proposed method are compared with those of the Fraunhofer approximation and those of the singular-value decomposition. 

The thirteenth paper introduces an algorithm to handle partial volume effect—a feature commonly seen in medical images, while segmenting a CT image to extract cerebrospinal fluid of ventricular region. The image histogram is used to compute global thresholds. The local thresholds for boundary pixels are then calculated based on neighboring pixels intensities. The combination of both global thresholding and local analysis refines the boundary of the ventricles. Also, a conditional probabilistic model is applied so that the algorithm becomes more robust. 

The fourteenth paper advances the authors' continuous studies on inverse problems of bioluminescence tomography. In real bioluminescence tomography experiments, besides the measurement error/noise in the usual sense of imaging analysis, the system errors induced by geometry mismatch, numerical and mathematical approximations are involved as well. A truncated total least squares method is proposed to treat both types of errors in equal footing. The residual-based error minimization is then conducted to determine model parameters. The effectiveness of the proposed algorithms is shown by numerical simulations with different noise levels and several physical settings. 

The fifteenth paper develops a general algorithm, that is, Shells and Spheres, to extract anatomical shape models from *n*-dimensional images. This algorithm creates a set of shells or spheres, centered at each image pixel and having maximized size, under a constraint that the boundary of shells should not cross the image edge. Variable scale statistics, such as intensity mean, variance, and first-order moment can be analyzed based on the constructed shells. The redundancy of the system can be reduced by examining only a subset of spheres which touch at least two spheres. The centers of these touched spheres are identified as medial. The extraction of medial ridges from images reveals underlying anatomical structures and can facilitate image segmentation. 

The sixteenth paper introduces a new class of Radon transform defined on a discontinuous line, having the shape of the letter V. Both analytical inverse transform and filtered back projection reconstruction procedure of the V-line Radon transform are established. These mathematical studies allow the two-dimensional image reconstruction from scattered radiation collected by a one-dimensional collimated camera. Numerical simulations validate the practical feasibility of the V-line Radom transform. 

The last paper focuses on the fast detection of eye vessel pixel from retinal images. The proposed vessel detection procedure consists of multiscale stages. The initial stage captures vessel wall pixels and sets up initial texture features for Gaussian-like 2D filtering. The fine structures of eye vessels are then analyzed in subsequent stages within the vessel body, and only selected regions that exhibit high homogeneity are treated with postconvolution so that the detection cost is quite low. Three vasculature cases are employed to illustrate the proposed algorithm. 

*Guowei Wei* *Guowei Wei* *Lalita Udpa* *Lalita Udpa* *Yang Wang* *Yang Wang* *Shan Zhao* *Shan Zhao*

